# Association between Risk Factors for Vascular Dementia and Adiponectin

**DOI:** 10.1155/2014/261672

**Published:** 2014-04-17

**Authors:** Juhyun Song, Won Taek Lee, Kyung Ah Park, Jong Eun Lee

**Affiliations:** ^1^Department of Anatomy, Yonsei University College of Medicine, 50 Yonsei-ro, Seoul 120-752, Republic of Korea; ^2^BK21 Plus Project for Medical Sciences and Brain Research Institute, Yonsei University College of Medicine, 50 Yonsei-ro, Seodaemun-gu, Seoul 120-752, Republic of Korea

## Abstract

Vascular dementia is caused by various factors, including increased age, diabetes, hypertension, atherosclerosis, and stroke. Adiponectin is an adipokine secreted by adipose tissue. Adiponectin is widely known as a regulating factor related to cardiovascular disease and diabetes. Adiponectin plasma levels decrease with age. Decreased adiponectin increases the risk of cardiovascular disease and diabetes. Adiponectin improves hypertension and atherosclerosis by acting as a vasodilator and antiatherogenic factor. Moreover, adiponectin is involved in cognitive dysfunction via modulation of insulin signal transduction in the brain. Case-control studies demonstrate the association between low adiponectin and increased risk of stroke, hypertension, and diabetes. This review summarizes the recent findings on the association between risk factors for vascular dementia and adiponectin. To emphasize this relationship, we will discuss the importance of research regarding the role of adiponectin in vascular dementia.

## 1. Introduction

Vascular dementia is the second most common type of dementia, accounting for 15 to 20% of all cases of dementia [1]. It is characterized by cognitive impairment and cerebrovascular pathologies [2]. According to the World Alzheimer Report 2011, an estimated 36 million people worldwide were afflicted with dementia. This number is increasing twofold every 20 years and will likely reach 115 million people by 2050 [3, 4]. Among the subtypes of dementia, vascular dementia is important because it results from a variety of causes, including cerebrovascular dysfunction. Vascular dementia and cerebrovascular diseases have common risk factors including hypertension, insulin resistance, diabetes, obesity, hyperhomocystinemia, and hyperlipidemia [5–8]. Recent clinical-pathological studies have focused on cognitive impairment and increased risk of dementia in patients with cerebrovascular disease [2, 9, 10]. In addition, vascular dementia is the most severe form of vascular cognitive impairment (VCI) [2, 11], and it results from subclinical vascular brain injury and stroke. VCI reflects the full range of cognitive alterations due to vascular factors [12]. A previous study demonstrates that reducing vascular risk factors inhibits cognitive decline progression [12]. Type 2 diabetes mellitus (T2DM), a risk factor for vascular dementia, is a heterogeneous metabolic disease characterized by reduced insulin sensitivity and relative insulin deficiency. T2DM and dyslipidemia frequently coexist with vascular dementia [13]. Adiponectin is almost exclusively secreted by adipocytes, and it appears to act as a modulator of anti-inflammation and insulin-sensitizer [14]. Adiponectin has beneficial effects on endothelial cells and affects the progression of stroke, atherosclerosis, and hypertension [15–20]. Plasma adiponectin levels are decreased in patients with cardiovascular disease and several metabolic disorders [21]. Several studies reported an inverse relationship between plasma adiponectin and T2DM [22–26]. In this review, we examine current research regarding the relationship between risk factors for vascular dementia and adiponectin.

## 2. Risk Factors for Vascular Dementia

**Figure 1:**
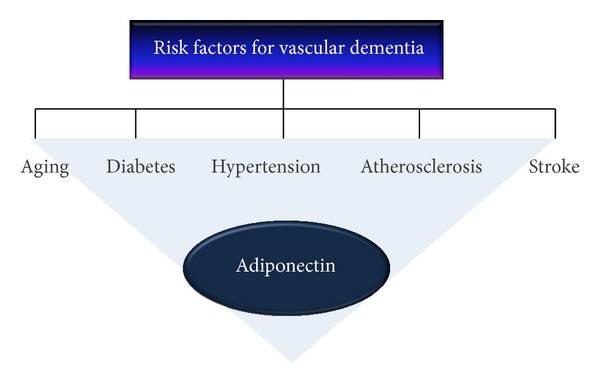
Risk factors for vascular dementia and adiponectin. Vascular dementia risk factors include aging, diabetes, hypertension, atherosclerosis, and stroke. Adiponectin is related to aging, diabetes, hypertension, atherosclerosis, and stroke by acting as a modulator or regulator in various mechanisms. Current researches have reported the role of adiponectin in diabetes, hypertension, atherosclerosis, and stroke.

Vascular dementia is regarded as the most severe form of VCI characterized by the presence of clinical stroke or vascular brain injury as well as cognitive impairment [2, 11, 27]. Several studies suggest that the risk factors for vascular dementia are almost identical as the risk factors for VCI. Common risk factors in both animal models and humans include hypertension, insulin resistance, hyperlipidemia, hyperhomocystinemia, atherosclerosis, and diabetes [28–32]. Age is also a risk factor for vascular dementia, suggesting that dementia in patients after the age of 65 increased gradually [33]. In addition, cerebrovascular dysfunction is a risk factor because the cerebrovascular function is reduced in patients with dementia [34–42]. In addition, another study suggests that metabolic syndrome, including insulin resistance, hypertension, and dyslipidemia, is associated with cognitive decline, a typical feature of vascular dementia [30].  Figure 1 shows that vascular dementia risk factors include aging, diabetes, hypertension, atherosclerosis, and stroke (Figure 1).

## 3. Adiponectin

Adiponectin is one of the most abundant adipokines [43, 44]. It has significant sequence similarities with complement factor C1q, whose protein is termed Acrp30 because it is a 30 kDa adipocyte complement-related protein [45, 46]. Adiponectin is the protein produced by adipose's most abundant gene transcript 1 (*APM1*) gene, and* APM1* gene is located on chromosome 3q27, a region associated with T2DM and metabolic syndrome susceptibility [47–50]. Several human genetic association studies emphasized that hypoadiponectinemia caused by the single nucleotides polymorphisms (SNPs) in* APM1* gene is important to investigate the role of adiponectin in a variety of diseases [14, 51–54] including insulin resistance, T2DM, and metabolic syndrome, such as obesity [55]. In white French subjects, 2 SNPs in the promoter region of* APM1* gene, SNP 11377 and SNP 11391, were strongly related to hypoadiponectinemia and T2DM [50]. In white German and North American subjects, the +276 G/T SNP was associated with obesity and insulin resistance [52, 56]. In Chinese subjects, the +276 G/T SNP was significantly involved in the coronary heart disease [57]. Adiponectin acts via binding its receptors, adiponectin receptor type 1 (AdipoR1) and type 2 (AdipoR2). AdipoR1 has a higher binding affinity to the globular form of adiponectin, whereas AdipoR2 has a higher binding affinity to full-length adiponectin [58]. Adiponectin binds to the C-terminal extracellular domain of AdipoR, and the N-terminal cytoplasmic domain interacts with APPL1 [59]. Adiponectin receptors are expressed in liver, hypothalamus, and brain vascular endothelial cells [60–62]. Adiponectin is associated with insulin resistance, obesity, T2DM, dyslipidemia, and cardiovascular diseases [63–70]. It is an effective insulin sensitizer [64, 71, 72], and it promotes peripheral insulin sensitivity [14] and inhibits liver gluconeogenesis [73]. Decreases in circulating adiponectin in the prediabetic state lead to insulin resistance [74]. Adiponectin activates AMP-activated protein kinase (AMPK), which activates insulin-independent glucose uptake by muscle, downregulates gluconeogenic enzymes, and increases muscle fatty acid oxidation [73]. Unlike other adipocyte-derived hormones, adiponectin gene expression and plasma concentration are inversely associated with body mass index (BMI) [75]. Reduced plasma adiponectin levels have also been reported in patients with coronary artery disease [19] as well as those with increased carotid intima media thickness [76]. Plasma adiponectin levels are inversely related to the platelet activation status of patients with cardiovascular risk factors [20]. Adiponectin suppresses platelet aggregation in hyperlipidemic rats by reversing the increase in inducible nitric oxide synthase expression while enhancing endothelial nitric oxide synthase activation [77, 78]. Current studies have reported the association between adiponectin and various diseases because adiponectin has multiple roles in glucose and lipid metabolisms and vascular system.

## 4. Adiponectin, Aging, and Diabetes

### 4.1. Aging, Insulin Signal Transduction, and Adipocytokines

Recently, the number of elderly patients with dementia has been increasing rapidly [79]. One epidemiology study suggests an exponential increase in the incidence of dementia after the age of 65, doubling roughly every 5 years, such that greater than 50% of centenarians are expected to suffer from dementia [33]. Aging induces an oxidative redox shift by attenuating mitochondrial metabolism and changing glycolysis metabolism [80]. These alterations initiate a damaging pathway involving signaling molecules, transcription factors, and epigenetic transcriptional regulators [80, 81]. Among several important pathways for maintaining longevity, insulin sensitivity has been considered a key factor for the healthy aging phenotype in humans [82, 83] and mice [84, 85]. Several studies have reported that insulin and insulin growth factor-1 (IGF-1) receptor regulate the lifespan of mice [86, 87]. In humans, growth hormone (GH) and IGF-1 deficiencies are also associated with life expectancy [88]. Insulin sensitivity normally decreases during aging, and the prevalence of metabolic syndrome (MetS) and insulin resistance substantially increases [89, 90]. In elderly persons, decreased insulin receptor (IR) levels and impaired insulin signaling have been observed predominantly in the hippocampus cortex and choroid plexus [81]. Impaired insulin receptor binding promotes chronic insulin resistance [91]. Muller et al. [92] reported that IGF-I signaling deteriorated in the brains of aged mice. This study demonstrated that activation of the brain IGF-1R/Akt/GSK-3*β* pathway was evidently reduced although older mice have higher brain IGF-1R levels [92]. In humans, insulin sensitivity decreases with aging and the prevalence of T2DM increases with advancing age [89, 90]. Reduced mitochondrial function contributes to decline in glucose uptake with advancing age and leads to insulin resistance [93–97]. IGF-1 concentrations decline with age and are associated with age-related changes in body composition by both increasing fat mass and decreasing muscle mass [98–100]. Aging alters the function and number of adipose cells which cause alterations in the secretion and function of the adipocytokines such as leptin and adiponectin [101]. A recent study demonstrated that cellular senescence of adipose tissue causes insulin resistance [102]. Considering these evidences, aging alters the function of adipose cells, and alteration in secretion of adipocytokines attenuates insulin sensitivity.

### 4.2. Adiponectin and Insulin Signal Transduction

Insulin and IRs are ubiquitously expressed in the brain [81, 103] where insulin can reach levels 10- to 100-fold greater than in plasma, particularly in the hippocampus, cortex, hypothalamus, olfactory bulb, and pituitary [81, 104]. IRs are largely localized in neurons and are less abundant in glia [103, 105]. Insulin produced by pancreatic *β*-cells is transported by cerebrospinal fluid (CSF) to the brain where it crosses the blood-brain barrier (BBB) [106, 107]. Similar to IRs, IGF-1Rs are widely distributed in the brain [107, 108]. Insulin/IGF-1-mediated activation of Akt leads to GSK-3*β* inactivation, which triggers multiple cascades, including synthesis of proteins involved in neuronal glucose metabolism and antiapoptotic mechanisms [104, 109]. Regarding brain glucose metabolism, recent studies suggest that changes in circulating insulin levels modulate glucose transporter (GLUT) expression [110, 111]. Cerebral IRs and IGF-1Rs are involved in cortical and hippocampal synaptic plasticity, thereby affecting memory and learning [105, 112]. In brain, insulin contributes to memory function through regulation of neurotransmitter receptors and synaptic function [113, 114]. Additionally, insulin signal transduction also promotes neurite outgrowth and axonal regeneration in the brain [105, 112, 115]. In the brain, insulin resistance results from perturbation of insulin signal transduction, causing systemic hyperglycemia. Decreased insulin and IGF-1 have been observed in Alzheimer's disease brain [116, 117]. Also, decreased insulin receptor substrate (IRS) protein levels related to insulin resistance [118] are associated with cognitive decline in dementia [119]. Impaired insulin transduction aggravates features of Alzheimer's disease including formation of neurofibrillary tangle caused by the decreasing brain glucose level and the increase of amyloid *β* aggregation [104, 106, 118, 120–122]. In addition, insulin resistance is closely linked with other metabolic symptoms, including hypertension and hyperlipidemia [123]. Adiponectin directly regulates glucose metabolism and insulin sensitivity. Adiponectin, via activation of AMPK and adiponectin, stimulates GLUT4 translocation and glucose uptake [124]. Adiponectin receptors activate AMPK, PPAR-*α*, and p38 MAPK to increase insulin sensitivity [58, 125]. An adaptor protein, APPL1, binds to adiponectin receptors that activate the AMPK and p38 MAPK pathways [126]. In addition, adiponectin decreases insulin resistance by decreasing triglyceride content in obese mice [127]. Increased tissue triglyceride content has been reported to interfere with insulin-stimulated phosphatidylinositol (PI) 3-kinase activation and subsequent GLUT 4 translocation and glucose uptake, thus leading to insulin resistance. Adipose tissue deficiency or lipodystrophy is associated with insulin resistance and metabolic dysregulation [128].* Adiponectin* knockout mice show impaired insulin secretion, and intravenous adiponectin injection into C57BL/6 mice induces insulin secretion [129, 130].* AdipoR1* and* 2* double knockout mice have increased triglyceride levels in the liver and exhibit insulin resistance and glucose intolerance, suggesting that AdipoR1 and AdipoR2 regulate lipid and glucose homeostasis [14, 131]. In conclusion, adiponectin and adiponectin receptors improve insulin resistance by modulating triglyceride level and impaired insulin signal transduction. Thus, regulation of adiponectin is important impaired insulin signal transduction to improve and also adiponectin may contribute to the improvement of cognitive decline in dementia.

### 4.3. Adiponectin, Diabetes, and Vascular Dementia

**Figure 2:**
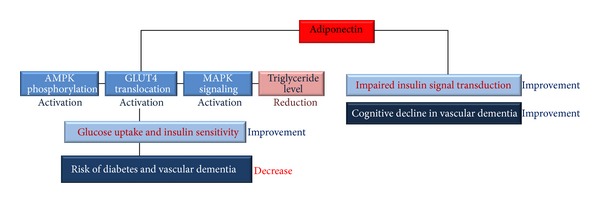
Adiponectin improves insulin sensitivity and reduces diabetes and vascular dementia risk. Adiponectin stimulates AMPK phosphorylation, GLUT4 translocation, and MAPK pathways. Adiponectin also reduces the levels of triglyceride. Consequentially, adiponectin increases glucose uptake and insulin sensitivity and reduces insulin resistance. In addition, adiponectin improves the cognitive decline by modulating impaired insulin signal transduction in vascular dementia brain. These mechanisms decrease the risk of diabetes and vascular dementia. Also, these mechanisms improve the memory dysfunction in dementia. AMPK: AMP-activated kinase, GLUT4: glucose transporter type 4, and MAPK: mitogen activated protein kinase.

Diabetes characterized by reduced insulin sensitivity is associated with thrombosis, myocardial infarction, and cerebrovascular disease, which can lead to infarctions and white matter ischemia [132]. Macrovascular disease causes approximately 80% of mortality in patients with T2DM. The risk of vascular diseases in patients with T2DM is decreased by lowering the blood pressure of patients with hypertension [133–135]. In addition, diabetes and hypoglycemia are associated with cognitive impairment [136–138]. Yaffe et al., in a 4-year prospective study, suggested that older women with impaired fasting glucose levels performed poorly on cognitive tests compared to those with normal glycemia [139]. Considering these associations, diabetes may be regarded as a risk factor of vascular dementia. Adiponectin levels are elevated in type I diabetics compared with healthy controls [140]. Several studies have consistently found that increased adiponectin levels are associated with reduced risk for T2DM [22, 25, 26]. Hypoadiponectinemia has been considered an underlying mechanism of insulin resistance in T2DM [141–145]. In cross-sectional studies, plasma adiponectin concentrations were significantly lower in patients with diabetes [146]. In a 5-year follow-up study of 1096 nondiabetics, the association between adiponectin and T2DM was attenuated after adjustment for homeostatic model assessment of insulin resistance (HOMA-IR) and was eliminated after adjustment for insulin sensitivity. These data suggest that the antidiabetic effect of adiponectin is due to insulin sensitization [147]. Adiponectin predicts against diabetes onset, and diabetic patients always show lower plasma adiponectin levels compared to the general population [148]. Thus, adiponectin reduces the risk of diabetes by regulating insulin signal transduction and insulin resistance. Suppression of adiponectin aggravates diabetes as a risk for vascular dementia.  Figure 2 shows that adiponectin stimulates the phosphorylation of AMPK and GLUT4 translocation and attenuates levels of triglyceride. As a result, adiponectin enhances glucose uptake and insulin sensitivity. This indicates that adiponectin reduces the risk of diabetes and vascular dementia (Figure 2).

## 5. Adiponectin, Hypertension, and Stroke

### 5.1. Adiponectin and Hypertension

**Figure 3:**
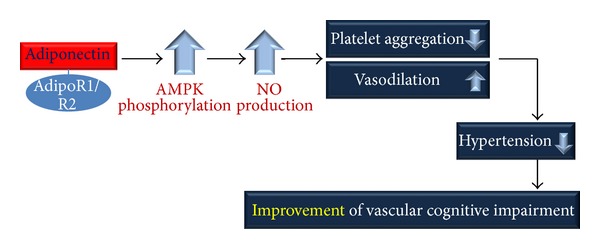
Adiponectin improves vascular cognitive impairment by stimulating NO production. Adiponectin, acting via AdipoR1 and AdipoR2, promotes AMPK phosphorylation and NO production. Increased NO reduces platelet aggregation and increases vasodilation. Consequentially, adiponectin decreases the risk of hypertension and improves vascular cognitive impairment. AMPK: AMP-activated kinase and NO: nitric oxide.

Hypertension has been reported as the most common risk factor for stroke worldwide and has also been gradually recognized as a risk factor for dementia [149]. Arterial hypertension contributes to the development and progression of cerebrovascular disease [150]. Hypertension exposes the cerebral microvasculature to pulsatile pressure and flow that cause vascular endothelium and smooth muscle cell tearing [151]. Many cross-sectional and longitudinal studies have demonstrated that dementia and VCI are associated with hypertension [152–156]. Therefore, previous studies suggest that hypertension is the most important risk factor for cerebral vessel dysfunction, and it contributes to cognitive decline [157, 158]. Pulse pressure (PP), a marker of arterial stiffness, has been connected with the risk of cognitive decline [159] and AD [160, 161]. Elevated pulse pressure increases the risk of cognitive decline and impaired language abilities [162]. Decreased blood pressure (BP) is a clinical manifestation of dementia in elderly subjects [163, 164]. Endothelial nitric oxide synthase (eNOS) and nitric oxide (NO) are crucial regulators of vascular homeostasis and, in particular, endothelial function [165, 166]. Endothelium-derived NO is a beneficial factor that promotes vasodilation and inhibits platelet aggregation, monocyte adhesion, and smooth muscle cell proliferation [167]. Adiponectin, acting via AdipoR1 and AdipoR2, promotes NO production through AMPK signaling pathway activation. AMPK activates eNOS through phosphorylation at Ser^1177^ and facilitates complex formation between eNOS and heat shock protein 90 (HSP-90), which is required for eNOS activation [167].* Adiponectin* knockout mice have reduced endothelial NO levels in vessel walls [168]. Adiponectin inhibits the inflammatory response and causes vasodilatation largely through AMPK/eNOS [169–172]. Adiponectin-induced AMPK signaling promotes phosphatidylinositol 3-kinase-Akt signaling, leading to angiogenic growth factor synthesis [170, 173]. A recent study also suggests that adiponectin inhibits vascular endothelial growth factor- (VEGF-) induced ROS generation and has an antioxidant role in the vasculature [174]. These actions of adiponectin are also mediated via inhibition of growth factor-stimulated extracellular signal regulated kinase (ERK) signaling. In addition, several studies indicate that adiponectin plays a role in the regulation of microvascular network flow and function [175, 176]. Some clinical research demonstrates that plasma adiponectin levels are positively associated with arterial vasodilation [177]. Considering the role of adiponectin in vascular function, decreased adiponectin raises the risk of hypertension.  Figure 3 shows that adiponectin increases AMPK phosphorylation and NO production. Platelet aggregation is decreased and vasodilation is increased due to NO production. Finally, adiponectin decreases the risk of hypertension and improves vascular cognitive impairment (Figure 3).

### 5.2. Adiponectin and Atherosclerosis

Atherosclerosis is a degenerative vessel disease that frequently affects large- to medium-sized arteries. In the brain, vessels of the circle of Willis are often involved [178]. Atherosclerotic plaques are prone to rupture with subsequent thrombosis [179, 180]. The thrombus resulting from plaque rupture leads to vessel occlusion or embolizes a smaller artery [181]. Atherosclerosis plaque rupture is related to inflammation, including secretion of cytokines and matrix-metalloproteinases, which are involved in vessel wall degradation [182–186]. Adiponectin plays the role of an antiatherogenic and anti-inflammatory modulator [18]. Several studies suggest that adiponectin inhibits many peptides and cytokines related to atherosclerosis progression [187]. Adiponectin inhibits monocyte adherence to TNF-*α*-stimulated endothelial cells by suppressing adhesion molecule expression [188, 189]. Adiponectin directly inhibits atherogenic molecules, such as intracellular adhesion molecule-1, vascular cellular adhesion molecule-1, and E-selectin, which are molecules associated with heightened leukocyte trafficking [189]. Adiponectin also attenuates expression of class A scavenger receptor in human macrophages and inhibits transformation of macrophages to foam cells [190]. The association between adiponectin and atherogenic factors indicates that regulation of adiponectin is important in atherosclerosis.

### 5.3. Adiponectin and Stroke

**Figure 4:**
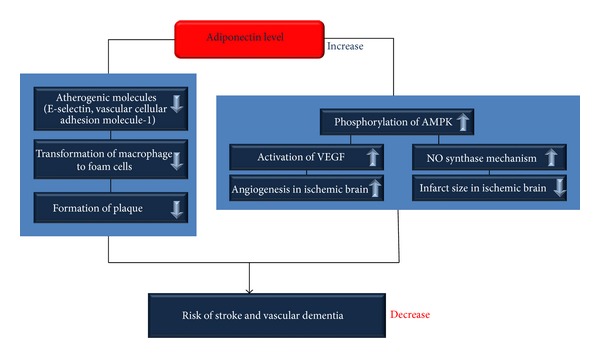
Adiponectin attenuates the risk of stroke and vascular dementia. Adiponectin decreases the expression of atherogenic molecules and formation of form cells in blood vessels. Adiponectin attenuates the risk of stroke by decreasing plaque formation in blood vessels. In addition, adiponectin binds with AdipoR1 and AdipoR2 and then activates the phosphorylation of AMPK. Increased AMPK phosphorylation promotes the activation of VEGF and NO synthase mechanism. As a result, adiponectin ameliorates angiogenesis in ischemic brain and reduces infarct size in ischemic brain. Consequentially, adiponectin decreases the risk of stroke and vascular dementia. AMPK: AMP-activated kinase, VEGF: vascular endothelial growth factor, and NO: nitric oxide.

Several studies reported that stroke doubles the risk for dementia (poststroke dementia), and approximately 30% of stroke patients develop cognitive dysfunction within 3 years [191–194]. An association between stroke and dementia is also observed in patients younger than 50 years, and up to 50% of these patients exhibit cognitive deficits after a decade [195]. One of the first population-based studies to assess the relationship between stroke and dementia was conducted in Rochester, Minnesota [196]. Many stroke patients show gradual but continuous deterioration after a single-stroke lesion. This deterioration is characterized clinically by cognitive and behavioral dysfunction [197]. Several cross-sectional and retrospective case-control studies have reported an association between low adiponectin levels and increased stroke risk [16, 198–201]. In addition, adiponectin levels are associated with coronary heart disease such as coronary vascular disease [202, 203]. Several studies demonstrate that hypoadiponectinemia increases the prevalence of coronary vascular disease [189, 204]. Several studies demonstrated that adiponectin knockout (APN-KO) mice showed severe injuries during cerebral ischemia-reperfusion [205, 206], while adiponectin injected APN-KO mice were reduced pathological ischemia-induced damage [207]. Adiponectin blocks the interaction between the endothelial cells and leukocytes in ischemia-reperfusion and also inhibits the secondary inflammation in cerebral ischemia-reperfusion [208]. Adiponectin reduced the infarct size through nitric oxide synthase dependent mechanism in cerebral ischemic stroke mice model [209, 210]. In addition, adiponectin activates AMPK phosphorylation in cerebral ischemic stroke mice model [211]. Then, the activation of VEGF by the activated AMPK signaling promotes angiogenesis in cerebral ischemic brain [212, 213]. Considering the results of the above studies, adiponectin is associated with the risk of stroke and reduces cerebral ischemia induced damage. This may be due to the roles of adiponectin as an antiatherogenic modulator and a vasodilator in vascular system.  Figure 4 shows that adiponectin decreases the expression of atherogenic molecules and plaque formation in blood vessels. Consequentially, adiponectin attenuates the risk of stroke and vascular dementia (Figure 4).

## 6. Conclusions

Risk factors for vascular dementia include advanced age, diabetes, hypertension, atherosclerosis, and stroke. Adiponectin, an adipokine, acts as an antidiabetic and antiatherogenic regulator. Insulin sensitivity is a key cellular mechanism related to diabetes, cerebrovascular dysfunction, and cognitive decline. Adiponectin is involved in insulin sensitivity, and increased adiponectin levels improve impaired insulin signaling. Moreover, adiponectin affects the cerebrovascular function by stimulating NO production and inhibiting transformation of macrophages to foam cells. Specifically, we summarize the findings as follows.

1. Vascular dementia characterized by cognitive decline is associated with increased age because insulin receptors, which are related to cognitive function, decrease with age. Adiponectin is associated with age-related diseases, including cardiovascular disease and metabolic disease. Adiponectin is mediated via the activation of AMPK, and adiponectin stimulates GLUT4 translocation and glucose uptake. Moreover, binding between adiponectin and adiponectin receptors activates AMPK, PPAR-*α*, and p38 MAPK to increase insulin sensitivity. In addition, in clinical studies, an association between decreased adiponectin and diabetes was demonstrated. In conclusion, adiponectin improves impaired insulin signaling and improves cognitive decline as a typical feature of vascular dementia.
2. Vascular dementia characterized by cerebrovascular dysfunction is associated with hypertension, atherosclerosis, and stroke. Adiponectin stimulates NO production through the AMPK signaling pathway. Adiponectin also plays the role of an antiatherogenic modulator. Adiponectin inhibits atherogenic molecules and attenuates the transformation of macrophages to foam cells. In conclusion, adiponectin improves vascular dysfunction and alleviates the progression of hypertension, atherosclerosis, and stroke as risk factors for vascular dementia.
3. Taken together, adiponectin attenuates the risk of vascular dementia and ameliorates vascular dementia-related pathologies including cerebrovascular dysfunction and cognitive decline which resulted from impaired insulin transduction and neuroinflammation.

In this review, we summarized the current research regarding the association between risk factors for vascular dementia and adiponectin. Considering the relationship between adiponectin and risk factors for vascular dementia including aging, diabetes, hypertension, atherosclerosis, and stroke, we suggest that further studies are necessary to examine the role of adiponectin in vascular dementia. Moreover, we emphasize that the regulation of adiponectin levels and receptors of adiponectin would be important for the prevention and treatment of vascular dementia.
